# Ethnobotanical Knowledge, Nutritional Composition, and Aroma Profile of *Vicia kulingiana* Bailey: An Underutilized Wild Vegetable Endemic to China

**DOI:** 10.3390/foods13060916

**Published:** 2024-03-18

**Authors:** Zhongxin Duan, Kai Mao, Xingxing Chen, Yiming Cui, Wei Wu, Jianbo Nie, Chunsong Cheng, Fengke Lin, Binsheng Luo

**Affiliations:** 1 Lushan Botanical Garden, Jiangxi Province and Chinese Academy of Sciences, Lushan 332900, China; duanzx@lsbg.cn (Z.D.); chenxx@lsbg.cn (X.C.); ymcui@lsbg.cn (Y.C.); chengcs@lsbg.cn (C.C.); 2 School of Chemistry and Chemical Engineering, Tianjin University of Technology, Tianjin 300384, China

**Keywords:** *Vicia kulingiana*, wild vegetable, nutritional composition, aroma compounds, ethnobotany, traditional knowledge

## Abstract

*Vicia kulingiana*, an endemic species, serves as a wild and underutilized vegetable traditionally consumed in China. However, ethnobotanical and chemical studies of this species are not available. This study analyzed its associated ethnobotanical knowledge, nutritional composition and aroma profile. Ethnobotanical surveys revealed its diverse traditional uses, especially as a nutritious vegetable. Further analysis showed *V. kulingiana* leaves to be high in protein, minerals, vitamin E, and dietary fiber. In total, 165 volatile compounds, such as terpenoids, alcohols, and ketones, were identified. Among them, *β*-ionone is the most abundant compound with a relative percentage of 8.24%, followed by 2,2,4,6,6-pentamethylheptane (3.2%), 3-(4-methyl-3-pentenyl)furan (2.37%), and linalool (1.68%). Results supported the traditional uses of *V. kulingiana*’s and highlighted its potential as a valuable food source, encouraging further research on its food applications. The documentation of ethnobotanical knowledge contributes to the conservation of this heritage.

## 1. Introduction

The genus *Vicia* L., a member of the Fabaceae family, holds a prominent position among resource plants. This taxonomic group primarily consists of herbaceous species, typically classified as annuals, biennials, or perennials [1]. *Vicia* species exhibit a widespread distribution across temperate regions in the Northern Hemisphere, extending into temperate zones in South America and East Africa [2,3]. This multifaceted genus comprises an extensive array of over 200 species, with the highest species diversity noted in Europe, the Caucasus region, and China, collectively acknowledged as the modern distribution centers for this genus [4,5]. In China, *Vicia* species show remarkable diversity, encompassing 43 species and 5 subspecies. They flourish predominantly in China’s Northwest, North, and Southwest regions, with comparatively fewer species found in South China, where only one species is documented [4,6].

*Vicia* plants are highly esteemed in agriculture, owing to their distinctive attributes, including their robust biomass production [7], brief growth cycles [8], elevated protein content, palatable nature [9], pleasant aroma, and efficient phosphorus absorption capabilities [10]. Consequently, they are widely utilized in agriculture, serving as premium forage, human dietary sources, and effective green manure [11,12]. Additionally, these species hold cultural and medicinal significance in local traditional healing practices [13,14]. Reports indicate that seeds of *Vicia* species encompass diverse constituents, including phenolic compounds, amino acids, lignans, and terpenoids [15,16]. However, research on the volatile components of *Vicia* species remains limited, with only one previous study investigating volatiles within this genus [17,18]. Romeo and Co-workers (2008) characterized 43 compounds in *Vicia sativa* L. leaf volatiles, including aliphatic hydrocarbons, alcohols, aldehydes, ketones, aromatic aldehydes, esters, monoterpenes, and sesquiterpenes, with (Z)-2-hexenal as the predominant aldehyde, using HS/SPME-GC/MS and GC-FID approaches [17].

*Vicia kulingiana* Bailey, belonging to the *Vicia* genus, is an endemic species in China, primarily distributed in the East [19]. This species is named after Guling Town (Kuling), as its type specimen was sourced from this locale, situated on the esteemed Mountain Lu Shan in Jiangxi Province, China. This species thrives in various habitats, ranging from valley bamboo forests, wetlands, and grasslands to sandy terrains, typically found at altitudes spanning from 200 m to 1200 m. Its flowering season is from April to August, followed by fruiting from June to September [19]. Our prior investigations in the Lu Shan region revealed that the tender leaves of *V. kulingiana* are a popular wild vegetable among locals, which has never been reported in other areas. This local popular practice is attributed to its distinctive fragrance, high nutritional value, and delightful taste, based on traditional botanical knowledge. However, the ethnobotanical importance and chemical analysis of *V. kulingiana* have yet to be comprehensively explored, leaving this potential food resource largely unutilized.

In the present investigation, a detailed examination was undertaken, focusing on the ethnobotany, nutritional components, and volatile compounds of *V. kulingiana*, with a particular emphasis on the foliar section of the plant. This research aims to document traditional uses associated with *V. kulingiana* and provides insight into its scientific basis from the perspective of nutrition and volatile aspects, which will be valuable for the preservation of associated knowledge and the utilization of this plant resource.

## 2. Materials and Methods

### 2.1. Ethnobotanical Survey

In conducting this ethnobotanical study on *V. kulingiana*, we employed a multifaceted approach that leveraged various literature databases, including Google Scholar (https://scholar.google.com/ accessed on 6 April 2023), PubMed (https://pubmed.ncbi.nlm.nih.gov/ accessed on 6 April 2023), and CNKI (https://www.cnki.net/ accessed on 6 April 2023). Additionally, we consulted sources, such as The Flora of China (http://www.efloras.org/ accessed on 6 April 2023) and local chronicles specific to the Lu Shan region, to gain comprehensive insights into the current state of research concerning this plant. Our primary objective centered on collecting data about *V. kulingiana*’s traditional uses.

Within the Lu Shan region of Jiangxi Province, we conducted ethnobotanical interviews focusing on *V. kulingiana* to delve into its local folk applications and utilization methods. The selected study locations encompassed villages and towns in or near Lu Shan. Our interviewees comprised elderly community members and restaurant proprietors, individuals renowned for their knowledge of the traditional uses of *V. kulingiana*. These interviews were conducted using a semi-structured interview format, predominantly addressing inquiries related to the plant’s local distribution, harvesting practices, processing techniques, and utilization. All interviewees were informed of the purpose of the interview and consent was obtained. Relevant interviews were also approved by the ethics department of Lushan Botanical Garden.

### 2.2. Plant Materials

Our experimental samples consisted of tender leaves of *V. kulingiana*, meticulously collected from Han Yang Peak (29°31′13.795″ N; 115°57′13.355″ E) in Guling Town, Lu Shan City, Jiangxi Province, China, in May 2023. The species was identified by the authors Xingxing Chen and Yiming Cui, who are proficient in botanical taxonomy. The plant voucher specimen (EB20230508001) was deposited in the Herbarium of Lushan Botanical Garden. Following collection, some of the fresh samples were used to conduct the nutrition test immediately, and the rest of the samples were preserved at −80 °C until they were ready for further analysis.

### 2.3. Nutritional Component and Total Flavonoid Evaluation

To assess the nutritional composition of *V. kulingiana* leaves, our study employed diverse analytical techniques encompassing the evaluation of macronutrients, amino acids, total flavonoids, vitamins, minerals, and other constituents. The analysis methods and detected items are listed in Table 1.

### 2.4. Volatile Compositions Using HS-SPME-GC-MS

The assessment of volatile metabolite components within *V. kulingiana* leaves was performed utilizing the Headspace Solid-Phase Microextraction (HS-SPME) technique coupled with an Agilent 8890 gas chromatograph (GC) and an Agilent 7000D mass spectrometer (MS) (Agilent, Palo Alto, CA, USA) in triplicates. Briefly, the samples were meticulously ground to obtain a fine powder immersed in liquid nitrogen. Subsequently, 500 mg of the obtained powder was promptly transferred into a 20 mL headspace vial containing a saturated NaCl solution to impede enzymatic reactions. Before the SPME analysis, each vial was subjected to a temperature of 60 °C for 5 min. Following this, a 120 µm DVB/CWR/PDMS fiber (Agilent) was introduced into the sample’s headspace, where it remained for 15 min at 60 °C. Upon the completion of sampling, the desorption of volatile organic compounds (VOCs) from the fiber coating occurred within the injection port of the GC apparatus at a temperature of 250 °C for 5 min in splitless mode. The VOCs were separated in a DB-5MS capillary column (30 m × 0.25 mm × 0.25 μm). Helium was used as the carrier gas at a 1.2 mL/min linear velocity. The injector temperature was maintained at 250 °C, while the detector was set at 280 °C. The oven temperature was programmed as follows: it was initiated at 40 °C (held for 3.5 min), ramped up at 10 °C/min to 100 °C, further increased at 7 °C/min to 180 °C, and finally raised to 25 °C/min to 280 °C, where it was held for 5 min. Mass spectra were recorded using electron ionization (EI) in ionization mode at 70 eV. The quadrupole mass detector, ion source, and transfer line temperatures were adjusted to 150 °C, 230 °C, and 280 °C, respectively.

The VOCs were identified using a widely targeted volatilomics (WTV) method recently described by Yuan et al. [31]. The features were characterized by comparing the deconvoluted spectra and Kovats’ retention index (RI) with those in the NIST 17 library and by comparing the determined RT and qualitative ions with those in the MWGCSIM1.0 library. The RI deviation was maintained within ±20. Two to three qualitative ions were selected for each compound. These ions were then detected separately for each group, adhering to the sequence of peak appearance. A compound was determined if the retention times of the detected peaks aligned with the standard reference and all the selected ions were present in the mass spectra of the samples after background subtraction [31]. The relative abundance of the annotated VOCs is expressed as a percentage of the peak area in the total ion chromatograms. The chemical structures, names, and aromas of VOCs were sourced from PubChem and Good Scents Company Information System. Additionally, odor identification was conducted by querying various databases, including an LRI and odor database (odour.org.uk accessed on 13 August 2023), Flavornet, and the human odor space database (flavornet.org accessed on 13 August 2023), as well as Flavor Ingredient Library (femaflavor.org accessed on 13 August 2023).

## 3. Results and Discussions

### 3.1. Ethnobotanical Knowledge of V. kulingiana

Based on the literature review, *V. kulingiana* is alternatively referred to as “Honghua Dou”, “Shanlu Dou”, and “Shan Can Dou” [19]. According to the documentation in Volume Two of the “Illustrated Handbook of Higher Plants in China” (1972) [32], it is noted that due to its rich content of minerals, amino acids, and proteins, *V. kulingiana* is frequently consumed as a wild vegetable; all parts of this plant can be utilized in traditional herbal medicine for their purported effects in clearing heat and detoxification.

Within the Lu Shan area, *V. kulingiana* is also recognized for its multifaceted uses, functioning both as a wild vegetable and a medicinal herb. All interviewees locally acknowledged its edibility and reported having experience consuming it. Moreover, more than half of the respondents preferred its distinct aroma. Due to its unique aroma and excellent palatability, local residents historically harvested its tender leaves for animal feed and as a primary source of vegetables during times of crop failure. Furthermore, according to the interview, its juice from the aerial part has been used in folk medicine to treat diarrhea and alleviate febrile convulsions in children, with notably positive therapeutic effects. Due to the introduction of modern Western medicine, few people are now aware of the medicinal properties of this plant, and almost no one is engaged in related practices anymore.

Nowadays, in the Lushan area, *V. kulingiana* is primarily employed as a wild, green vegetable for entertaining guests, although it is relatively scarce in the market due to its limited availability. These ethnobotanical records may reflect several key aspects: (1) the long history of safe consumption of tender *V. kulingiana* leaves, (2) the notable nutritional content of these leaves, and (3) that it might contain unique aromatic compounds that many local people are fond of.

**Figure 1:**
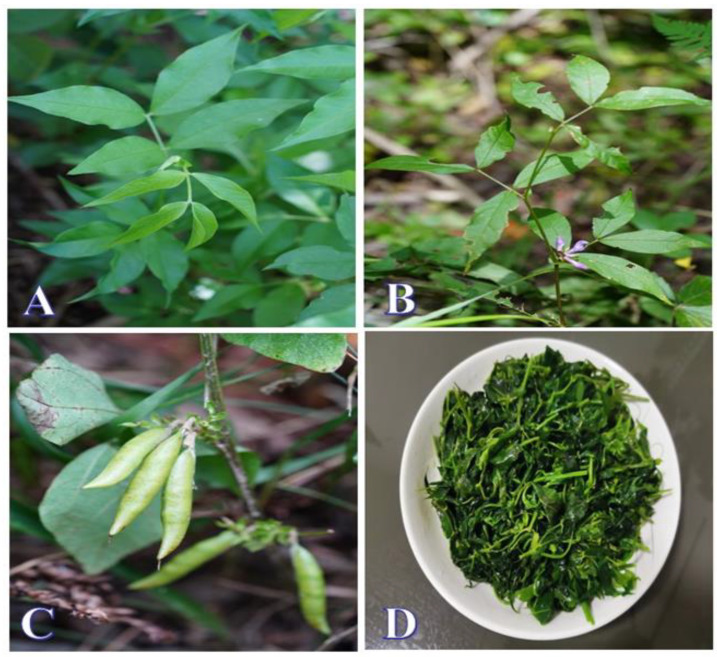
*V. kulingiana*: natural state and cooked preparation. (**A**) Plant; (**B**) flowers; (**C**) fruits; (**D**) fried *V. kulingiana*.

According to our field trips, *V. kulingiana* demands highly specific habitat conditions concerning soil, climate, and altitude during its growth, so its wild distribution in the Lu Shan region is mainly concentrated around Han Yang Peak. This coincided with the growing season, allowing us, guided by local experts, to successfully collect *V. kulingiana* (as depicted in Figure 1A–C). Fortunately, we also had the privilege of fully participating in and documenting the entire process of transforming its tender leaves into a prepared dish. Typically, local residents gather the tender leaves of *V. kulingiana*, wash them thoroughly, briefly blanch them in hot water, and then stir-fry them in rapeseed oil until they are ready for consumption (as illustrated in Figure 1D).

### 3.2. Analysis of Nutritional Components and Total Flavonoids in V. kulingiana Leaves

In this study, we determined the levels of energy, protein, fat, carbohydrates, dietary fiber, minerals, amino acids, vitamins, and flavonoids in *V. kulingiana* leaves (Table 2).

#### 3.2.1. Basic Nutritional Components

The tender leaves of *V. kulingiana* contain approximately 60.9 kcal/100 g of energy, which is notably higher than that contained in many other vegetable foods. For comparison, green beans contain around 27 kcal/100 g, while pumpkins provide only 22 kcal/100 g [35]. Although its energy content is similar to that of most starchy vegetables, such as potatoes (76 kcal/100 g) and sweet potatoes (99 kcal/100 g), it is considerably lower than that of typical grains like wheat (317 kcal/100 g) and millet (358 kcal/100 g) [36]. Also, the carbohydrate content in *V. kulingiana* leaves is moderate, with 1.4 g/100 g of starch content, which is lower than that of grains [35]. Although this plant has a lower energy or carbohydrate content compared with that of some grains, its nutritional value cannot be overlooked, supporting its traditional use as a wild vegetable or animal feed, especially in times of scarcity.

*V. kulingiana* leaves are notably protein-rich, containing 8.73 g per 100 g of edible portion. Its protein content is significantly higher than that of most leafy green vegetables, such as green beans (2.5 g/100 g of edible portion) and mung bean sprouts (2.1 g/100 g), and it is even close to the protein content of wheat, a grain known for its protein content, which stands at 11.9 g/100 g [35]. As a result, *V. kulingiana* leaves, being leafy greens, offer a substantial source of protein and can contribute to daily protein intake for humans.

Dietary fiber, a type of non-starch polysaccharide usually contained in vegetables, fruits, and whole grains, provides various health benefits, including a reduced risk of heart disease and type 2 diabetes [36]. The total dietary fiber content in *V. kulingiana* leaves is approximately 4.82 g/100 g, with the majority being insoluble dietary fiber (4.255 g/100 g) and only 0.565 g/100 g being soluble dietary fiber. While the dietary fiber content in *V. kulingiana* leaves is lower than that in common grains, such as wheat (10.8 g/100 g) and bran (31.3 g/100 g) [35], the recommended daily intake of dietary fiber in China is approximately 27 g per day [34]. This suggests that *V. kulingiana* leaves may not be the primary source of dietary fiber.

In summary, *V. kulingiana* leaves are rich in energy nutrients and protein, making them a suitable dietary option for meeting human nutritional needs. They possess significant potential as a valuable vegetable for consumption. The series of results from the nutritional content test also prove the scientific validity of traditional knowledge of the utilization of this plant.

#### 3.2.2. Trace Elements

Wild edible plants have long been an excellent source of trace elements, and their consumption contributes to meeting nutritional requirements while overcoming micronutrient deficiencies at minimal costs [37,38]. The current study also measured the mineral content in *V. kulingiana* leaves, as shown in Table 2.

*V. kulingiana* leaves are remarkably rich in several trace elements, including copper, potassium, phosphorus, and calcium. The copper content is particularly noteworthy at 2.6 mg/100 g, its potassium content reaches 3650 mg/100 g, its phosphorus content is 1110 mg/100 g, and its calcium content is 565 mg/100 g. To meet the recommended daily intake of these minerals for the average adult, one would only need to consume approximately 31 g, 55 g, 65 g, and 142 g of *V. kulingiana* leaves, respectively. Furthermore, iron and zinc contents are also abundant, with 100 g of *V. kulingiana* leaves meeting the daily iron and zinc requirements for an individual. For comparison, spinach, known for its iron content, contains approximately 2.9 mg of iron per 100 g of edible portion [35], whereas *V. kulingiana* leaves contain 22 mg. Similarly, oysters, a zinc-rich food, contain 9.39 mg of zinc per 100 g, while beef contains only about 4 mg/100 g, yet *V. kulingiana* leaves provide 11.5 mg/100 g of zinc. Nuts, in general, are known for their magnesium content, with almonds being one of the highest sources, containing around 178 mg of magnesium per 100 g. Remarkably, *V. kulingiana* leaves contain 360 mg of magnesium [35], surpassing the content of that in almonds. Additionally, the sodium content in *V. kulingiana* leaves is relatively moderate. The leaves of *V. kulingiana* are rich in mineral elements, especially some that are relatively difficult to obtain from regular foods, such as calcium, copper, iron, zinc, magnesium, and more. Their content far exceeds that in other common vegetables, requiring only a small intake to meet the human body’s nutritional needs.

#### 3.2.3. Functional Nutrients

Regarding vitamins, *V. kulingiana* leaves exhibit deficient levels of vitamins A, B1, B2, and C. The content of vitamin B2 is only 0.319 mg/100 g, and to meet the human body’s requirements, supplementation from other vegetables and fruits is necessary. However, *V. kulingiana* leaves exhibit a remarkably high vitamin E content, reaching 2220 mg/100 g. The highest vitamin E content in vegetables, represented by dehydrated Chinese cabbage, is only 187 mg/100 g [35]. Vitamin E, a fat-soluble vitamin, primarily functions as an antioxidant, eliminating free radicals that may harm human cells. It holds the potential for promoting health and preventing or treating diseases [39]. Therefore, *V. kulingiana* leaves serve as an excellent natural source of vitamin E.

#### 3.2.4. Total Flavonoids

Flavonoid compounds are found in fruits and vegetables and have demonstrated various potential beneficial activities in animal studies and human trials, such as antioxidant, antimicrobial, and cardiovascular disease-fighting properties [40]. Test results reveal that *V. kulingiana* leaves contain 160 mg/100 g of flavonoids, which is significantly higher than the concentration of those in typical fruits and vegetables, even surpassing the concentration found in dried tea leaves per unit [35]. This indicates that *V. kulingiana* leaves likely possess considerable potential for dietary supplementation and medicinal use. However, it is important to note that this study only examined the total flavonoid content and did not isolate, extract, or identify specific flavonoid compounds. Future research on *V. kulingiana* leaves should involve a comprehensive identification of flavonoid types in order to explore their full functional potential.

### 3.3. Analysis of Volatile Components in V. kulingiana Leaves

Using HS-SPME-GC-MS technology, in total,165 VOCs were characterized in *V. kulingiana* leaves, representing 79.83% of the total volatile content. These compounds mainly include terpenoids, alcohols, carboxylic acids, heterocyclic compounds, aromatic hydrocarbons, and ketones (Table S1). The main compounds with relative contents over 1% are shown in Table 3. Among them, the terpenoids of *β*-ionone (8.24%), 3-(4-methyl-3-pentenyl) furan (2.37%), and linalool (1.68%) are known for their floral and citrus-like aromas, which may play a crucial role in the fragrance of *V. kulingiana* [41,42,43]. Additionally, there is a significant presence of heterocyclic compounds, such as dihydroactinidiolide and 2-methyl-3-furanthiol, with a relative content of 1.55% and 1.35%, which could contribute a meaty or fishy taste to *V. kulingiana* [44,45]. Furthermore, ketone compounds like 2,6,6-trimethylbicyclo[3.2.0]hept-2-en-7-one can potentially enhance the sweet aroma of *V. kulingiana* [46]. Based on these findings, we speculate that the rich terpenoid and alcohol compounds in *V. kulingiana* leaves might be the primary sources of their distinctive fragrance. The presence of these volatile components reveals the material basis for the unique aroma of this wild vegetable, further validating that the related traditional knowledge has a certain scientific basis.

### 3.4. Food Safety

Based on a literature review and field investigations, it is evident that in the past, *V. kulingiana* leaves from Guling were commonly used as both wild vegetables and animal feed. Nutritional analysis results demonstrated that *V. kulingiana* leaves are rich in nutrients, displaying excellent protein content, diverse trace elements, and their respective quantities. The traditional practice of using them as wild vegetables and animal feed holds scientific merit. Furthermore, the high content of vitamin E and flavonoids in *V. kulingiana* leaves suggests their significant potential for medicinal use. According to Chinese plant records, the entire *V. kulingiana* plant can be used for medicinal purposes, and further exploration is warranted to understand its underlying principles and therapeutic effects.

The analysis of volatile components has revealed that *V. kulingiana* leaves contain a rich variety of volatile organic compounds, with as many as 165 different types, each contributing to distinct aromas (as shown in Table 3). Additionally, the relative content of each component is relatively low, with the highest content, of *β*-Ionone, only accounting for 8.24% of the total. This complexity in their aroma composition suggests that it is insufficient to draw definitive conclusions solely based on the relative content or proportion of volatile substances. Further experimental research is needed to confirm the contribution of volatile compounds to the aroma of *V. kulingiana* leaves.

Furthermore, the safety of *V. kulingiana* leaves has not yet been verified. While no toxic or harmful substances were found among the volatile organic compounds, assessing the safety risks of consuming this wild plant solely based on the results of volatile components testing is insufficient. The following considerations should be taken into account: (1) Volatile components cannot represent all the ingested substances in *V. kulingiana* leaves, and non-volatile natural toxic components such as alkaloids may be present and require additional testing. (2) Safety assessment should not rely solely on chemical composition; the specific content of each component must also be considered, as even aromatic volatile compounds can exhibit toxicity at low doses. (3) Different individuals may have varying tolerances to the same food components, potentially leading to allergies or unexpected reactions. (4) Besides components, factors such as processing techniques and storage conditions can also affect food safety. Therefore, if there are plans for future development and utilization, such as the development of new foods or feeds, toxicity studies are indispensable.

## 4. Conclusions

This study comprehensively analyzed the ethnobotanical knowledge, nutritional value, and aroma profile of the wild vegetable *V. kulingiana* in China. Results demonstrated its high nutritional content and complex mix of volatile compounds, supporting its potential as a nutritious and flavorful food source, pending further safety studies. Additional research on its flavonoids, medicinal uses, and agricultural development is warranted to promote the conservation and utilization of this valuable underutilized plant species. Findings contribute to the ethnobotanical record of *V. kulingiana* and provide a scientific basis on which to inform its future application in medicine, agriculture, and food science.

## Figures and Tables

**Table 1:** Determination of nutritional components in *V. kulingiana*.

Test Items		Determination Methods
Macronutrient	Energy	GB/Z 21922-2008 [20]
	Protein	GB 5009.5-2016 method 1 [21]
	Fat	GB 5009.6-2016 method 2 [22]
	Dietary fiber	GB 5009.88-2014 [23]
	Starch	GB 5009.9-2016 method 2 [24]
	Trace elements	GB 5009.268-2016 method 2 [25]
Vitamins	Vitamin A	GB 5009.82-2016 method 1 [26]
	Vitamin B1	GB 5009.84-2016 method 2 [27]
	Vitamin B2	GB 5009.85-2016 method 1 [28]
	Vitamin C	GB 5009.86-2016 method 2 [29]
	Vitamin E	GB 5009.82-2016 method 1 [26]
Total flavonoids		ISO 20759:2017 (Annex C) [30]

The determined methods mostly followed the Standardization Technical Guidance Document of the People’s Republic of China and International Organization for Standardization.

**Table 2:** The result of the nutrition and total flavonoids tests.

Test Items		Test Results	Reference Intake Amount Daily [33,34]
Macronutrients (g/100 g)	Energy	255 kJ (60.9 kcal)	——
	Protein	8.73	55–65 g/d
	Fat	0.9	770–997.5 kcal (/kg·d)
	Soluble dietary fiber	0.565	——
	Dissoluble dietary fiber	4.255	——
	Total dietary fiber	4.82	27 g
	Starch	1.4	120–150 g/d (carbohydrates)
Minerals (mg/100 g)	Cu	2.6	0.8 mg/d
	Fe	22	12–20 mg/d
	Zn	11.5	7.5–12.5 mg/d
	Ca	565	800 mg/d
	Mg	360	330 mg/d
	K	3650	2000 mg/d
	Na	16.5	1500 mg/d
	P	1110	720 mg/d
Vitamins (mg/100 g)	Vitamin A	undetected	700–800 μg/d
	Vitamin B1	undetected	1.2–1.4 mg/d
	Vitamin B2	0.319	1.2–1.4 mg/d
	Vitamin C	undetected	100 mg/d
	Vitamin E	2220	14 mg/d
Total flavones (mg/100 g)		160	——

**Table 3:** Main volatile components of *V. kulingiana* leaves analyzed via HS-SPME-GC-MS.

No.	Formula	Compounds	Types	RI	Odor	I%
1	C_13_H_20_O	*β*-Ionone	Terpenoids	1491	floral, woody, sweet, fruity, berry, tropical, beeswax	8.24
2	C_12_H_26_	2,2,4,6,6-Pentamethylheptane	Hydrocarbons	990		3.2
3	C_10_H_14_O	3-(4-Methyl-3-pentenyl) furan	Terpenoids	1101	woody	2.37
4	C_10_H_18_O	Linalool	Terpenoids	1101	floral, green	1.68
5	C_10_H_14_O	2,6,6-Trimethylbicyclo[3.2.0]hept-2-en-7-one	Ketone	1108		1.68
6	C_10_H_14_	(3*E*,5*E*)-2,6-Dimethyl-1,3,5,7-octatetraene	Hydrocarbons	1131		1.66
7	C_11_H_16_O_2_	Dihydroactinidiolide	Heterocyclic compound	1532	sulfury, meaty, fishy, metallic	1.55
8	C_10_H_14_O	(1-Methoxypropyl)benzene	Aromatics	1104		1.52
9	C_11_H_15_Br	(5-Bromopentyl)benzene	Aromatics	1487		1.43
10	C_5_H_6_OS	2-Methyl-3-furanthiol	Heterocyclic compound	870	sulfury, meaty, fishy, metallic	1.35
11	C_6_H_10_N_4_	3,5-Dimethyl-1*H*-pyrazole-1-carboximidamide	Heterocyclic compound	1096		1.28
12	C_6_H_14_N_2_	1-Methylpyrrolidine-2-methylamine	Heterocyclic compound	1104		1.23
13	C_14_H_22_	1-Nonen-4-ol	Alcohol	1485		1.17
14	C_9_H_18_O	8,8-Dimethyl-9-methylene-1,5-cycloundecadiene	Hydrocarbons	1103		1.17
15	C_8_H_16_O	3-Cyclopentyl-1-propanol	Alcohol	1102		1.06
16	C_8_H_7_NO	1,3-Dihydro-2*H*-indol-2-one	Heterocyclic compound	1478		1.04

RI: Retention index of compounds on non-polar chromatographic column. Odor describes the substance’s aroma. I indicates the relative abundance of detected VOCs.

## Data Availability

The original contributions presented in the study are included in the article/Supplementary Materials, further inquiries can be directed to the corresponding authors.

## Supplementary Materials

The following supporting information can be downloaded at https://www.mdpi.com/article/10.3390/foods13060916/s1: Table S1: Volatile components of *V. kulingiana* leaves analyzed by HS-SPME-GC-MS.
