# Supplementary material for: Ethnobotanical Knowledge, Nutritional Composition, and Aroma Profile of Vicia kulingiana Bailey: An Underutilized Wild Vegetable Endemic to China
Source: Foods. 2024 Mar 18;13(6):916. doi: 10.3390/foods13060916 (PMC10970078; doi:10.3390/foods13060916)
Supplement: Supplementary file 1 [file foods-13-00916-s001.zip › foods-2917216-supplementary.pdf]

Table S1 Volatile components of *V. kulingiana* leaves analyzed by HS-SPME-GC-MS

No.	Formula	Compounds	Types	RI	Odor	I(%)
1	$C_{13}H_{20}O$	*β*-Ionone	Terpenoids	1491	floral, woody, sweet, fruity, berry, tropical, beeswax	8.24
2	$C_{12}H_{26}$	2,2,4,6,6-Pentamethylheptane	Hydrocarbons	990		3.2
3	$C_{10}H_{14}O$	3-(4-Methyl-3-pentenyl)furan	Terpenoids	1101	woody	2.37
4	$C_{10}H_{18}O$	Linalool	Terpenoids	1101	floral, green	1.68
5	$C_{10}H_{14}O$	2,6,6-Trimethylbicyclo[3.2.0]hept-2-en-7-one	Ketone	1108		1.68
6	$C_{10}H_{14}$	(3*E*,5*E*)-2,6-Dimethyl-1,3,5,7-octatetraene	Hydrocarbons	1131		1.66
7	$C_{11}H_{16}O_2$	Dihydroactinidiolide	Heterocyclic compound	1532	musky, coumari n	1.55
8	$C_{10}H_{14}O$	(1-Methoxypropyl)benzene	Aromatics	1104		1.52
9	$C_{11}H_{15}Br$	(5-Bromopentyl)benzene	Aromatics	1487		1.43
10	C_5H_6OS	2-Methyl-3-furanthiol	Heterocyclic compound	870	sulfury, meaty, fishy, metallic	1.35
11	$C_6H_{10}N_4$	3,5-Dimethyl-1*H*-pyrazole-1-carboximidamide	Heterocyclic compound	1096		1.28
12	$C_6H_{14}N_2$	1-Methylpyrrolidine-2-methylamine	Heterocyclic compound	1104		1.23
13	$C_9H_{18}O$	1-Nonen-4-ol	Alcohol	1103		1.17
14	$C_{14}H_{22}$	1,5-Cycloundecadiene, 8,8-dimethyl-9-methylene-	Hydrocarbons	1485		1.17
15	$C_8H_{16}O$	3-Cyclopentyl-1-propanol	Alcohol	1102		1.06
16	C_8H_7NO	1,3-Dihydro-2*H*-indol-2-one	Heterocyclic compound	1478		1.04
17	$C_{15}H_{24}$	Eremophilene	Terpenoids	1494		0.96
18	$C_{15}H_{24}$	.Alpha.-muurolene	Terpenoids	1499	woody	0.94
19	$C_{15}H_{24}$	1-Methyl-4-(6-methylhept-5-en-2-yl)cyclohexa-1,3-diene	Terpenoids	1482		0.91
20	$C_8H_8O_3$	1-(2,4-Dihydroxyphenyl)ethanone	Ketone	1538		0.87
21	$C_6H_{12}O$	(*Z*)-Hex-3-en-1-ol	Alcohol	856	fresh, green, grass, foliage, vegetabl	0.86

					e, herbal, oily	
22	$C_{15}H_{24}$	5-(1,5-Dimethyl-4-hexenyl)-2-methyl-1,3-cyclo hexadiene	Terpenoids	1495	spice, fresh, sharp	0.84
23	$C_{15}H_{22}$	1-Methyl-4-(6-methylhept-5-en-2-yl)benzene	Terpenoids	1483	herbal	0.84
24	$C_{15}H_{22}$	Eudesma-2,4,11-triene	Terpenoids	1479		0.84
25	$C_4H_5N_3$	Iminodiacetonitrile	Nitrogen compounds	1108		0.83
26	$C_{10}H_{16}O$	Carvenone	Terpenoids	1257	spearmi nt	0.82
27	$C_{10}H_{16}O$	6-Isopropyl-3-methylcyclohex-2-enone	Terpenoids	1261	herbal, minty, camphor , medicin al	0.82
28	$C_8H_{11}N$	Benzeneethanmine	Amine	1102	ammoni acal, fishy	0.8
29	$C_8H_{14}O_3$	Butanoic acid, anhydride	Others	1120	buttery	0.77
30	$C_8H_{14}O_3$	Hexanoic acid, 3-oxo-, ethyl ester	Ester	1120	fruity, pineappl e, green, sweet, licorice, vanilla	0.77
31	$C_{10}H_{16}O$	Fenchone	Terpenoids	1096	herbal, cedar leaf, bitter, thuja, camphor , earthy, woody	0.72
32	$C_8H_{18}O$	3-Octanol	Alcohol	994	earthy, mushro om, herbal, melon, citrus, woody, spicy,	0.69

33	C_6H_8S	3-Ethyl-thiophene	Heterocyclic compound	869	minty styrene	0.64
34	$C_7H_{12}O_2$	2,2-Dimethyl-3-pentenoic acid	Acid	997		0.63
35	$C_{10}H_{18}O$	2-Methyl-6-methylene-7-octen-4-ol	Terpenoids	1097		0.62
36	$C_6H_{13}N$	CyclohexylAmine	Amine	862		0.6
37	$C_{10}H_{16}$	1,6-Dimethyl-1,5-cyclooctadiene	Hydrocarbons	1103		0.6
38	$C_{11}H_{24}$	Undecane	Hydrocarbons	1100	alkane	0.58
39	$C_8H_{16}O$	1-Octen-3-ol	Alcohol	980	fatty, fruity, grassy, mushroom, perfumy, sweet	0.58
40	$C_9H_{16}O_2$	(*Z*)-Hex-3-en-1-yl propionate	Ester	1100	green, fresh, fruity, apple, pear, vegetable, melon, banana, peach	0.56
41	$C_4H_7N_3O$	1-(2-Hydroxyethyl)-1,2,4-triazole	Heterocyclic compound	1095		0.55
42	$C_{11}H_{20}$	1-Undecyne	Hydrocarbons	1095		0.54
43	$C_{10}H_{16}O$	2,7,7-Trimethyl-3-oxatricyclo[4.1.1.02,4]octane	Terpenoids	1103	green	0.53
44	$C_5H_{11}NS_3$	*N*,*N*-Dimethyl-1,2,3-trithian-5-Amine	Heterocyclic compound	1491		0.52
45	$C_8H_{10}O$	(4-Methylphenyl)methanol	Alcohol	1106	mild, floral	0.49
46	C_7H_8O	2-Methylphenol	Phenol	1053	phenol	0.48
47	$C_8H_{14}O_2$	5-Butyldihydrofuran-2(3*H*)-one	Ester	1261	sweet, coconut, waxy, creamy, tonka, dairy, fatty	0.46
48	$C_{15}H_{32}$	Pentadecane	Hydrocarbons	1499	waxy	0.46
49	$C_{13}H_{20}O$	1,1,4*A*-Trimethyl-3,4,4*a*,5,6,7-hexahydronaphthalen-2(1*H*)-one	Ketone	1491		0.44

50	$C_{11}H_{24}$	5-Methyl-decane	Hydrocarbons	1057		0.41
51	$C_{15}H_{24}$	Beta-eudesmene	Terpenoids	1486	herbal	0.41
52	$C_6H_8O_2$	5-Ethyl-2(5*H*)-furanone	Heterocyclic compound	966	spice	0.41
53	$C_{10}H_{18}O$	Isoborneol	Terpenoids	1170	pine, woody, camphor	0.41
54	$C_{10}H_{18}O$	Endo-borneol	Terpenoids	1170	pine, woody, camphor, balsamic	0.41
55	$C_6H_{12}O_2$	Isobutyl acetate	Ester	773	sweet, fruity, ethereal, banana, tropical	0.4
56	$C_{17}H_{36}$	2,6,10-Trimethyl-tetradecane	Hydrocarbons	1539		0.4
57	$C_8H_7NO_3$	7-Hydroxy-6-methylfuro[3,4-*c*]pyridin-1(3*H*)-one	Heterocyclic compound	1484		0.4
58	$C_6H_{10}O$	Trans-2-hexenal	Aldehyde	853	sweet, almond, fruity, green, leafy, apple, plum, vegetable	0.4
59	$C_6H_{10}O$	(*E*)-Hex-2-enal	Aldehyde	853	green, grassy	0.4
60	$C_{10}H_{18}O$	7-Methyl-3-methylene-6-octen-1-ol	Terpenoids	1195		0.39
61	$C_{13}H_{28}$	5,7-Dimethylundecane	Hydrocarbons	1190		0.39
62	$C_8H_8O_2$	Benzoic acid, methyl ester	Ester	1098	phenol, wintergreen, almond, floral, canga	0.39
63	$C_{15}H_{32}$	4-Methyltetradecane	Hydrocarbons	1459		0.37
64	$C_{13}H_{28}$	2,3,5-Trimethyldecane	Hydrocarbons	1411		0.37
65	$C_{10}H_{14}O$	2,6,6-Trimethylcyclohexa-1,3-dienecarbaldehyde	Terpenoids	1206	fresh, herbal, phenol,	0.36

66	C_8H_8O	BenzeneacetAldehyde	Aldehyde	1046	metallic, rosemary, tobacco, spicy floral, honey, rose, cherry	0.36
67	$C_{10}H_{14}O$	2,6,6-Trimethyl-2,4-cycloheptadien-1-one	Terpenoids	1243	minty	0.36
68	$C_{14}H_{30}$	4,6-Dimethyldodecane,	Hydrocarbons	1325		0.36
69	$C_{14}H_{30}$	Tetradecane	Hydrocarbons	1400	mild, waxy	0.36
70	$C_{10}H_{20}O$	Levomenthol	Terpenoids	1177	minty	0.34
71	$C_{10}H_{14}$	1-Butylbenzene	Aromatics	1054		0.33
72	$C_4H_4N_2$	Pyrazine	Heterocyclic compound	726	pungent, sweet, corn, roasted, hazelnut, barley	0.33
73	$C_{13}H_{28}$	3-Methyl-5-propylnonane	Hydrocarbons	1185		0.33
74	$C_{10}H_{18}O$	Tetrahydro-4-methyl-2-(2-methyl-1-propenyl)-2*H*-pyran	Terpenoids	1113	sweet, floral, aromatic, rose, fresh, bay, leafy	0.31
75	$C_5H_6S_2$	2-Thiophenemethanethiol	Heterocyclic compound	1105	roasted, coffee, fishy	0.31
76	$C_{10}H_8O_2$	3-Methyl-2*H*-chromen-2-one	Heterocyclic compound	1490		0.3
77	$C_{10}H_{16}O$	(*Z*)-(3,3-Dimethylcyclohexylidene)acetaldehyde	Aldehyde	1226		0.3
78	$C_9H_{12}O_2$	3,4-Dimethoxytoluene	Aromatics	1233		0.3
79	$C_{10}H_{18}O$	3,7-Dimethyl-3*Z*,6-octadien-1-ol	Terpenoids	1240		0.3
80	$C_6H_{10}O_2$	5-Ethyldihydrofuran-2(3*H*)-one	Ester	1055	sweet, caramel	0.3
81	$C_7H_{10}O_3$	5-Ethyl-3-hydroxy-4-methylfuran-2(5*H*)-one	Heterocyclic compound	1195	sweet, fruity, caramel, maple,	0.3

					fenugreek, brown, sugar, nutty, chicory, praline, butterscotch	
82	$C_9H_{12}O_2$	2-Acetyl-4,4-dimethyl-cyclopent-2-enone	Ketone	1213		0.3
83	$C_{12}H_{16}O_3$	2-Phenoxyethyl isobutyrate	Ester	1488	green, fruity, waxy, apple, nuances	0.29
84	$C_{10}H_{16}O$	(2*Z*)-3,7-Dimethylocta-2,6-dienal	Terpenoids	1240	sweet, citral, lemon, peel	0.29
85	$C_8H_{17}N$	N-Allyl-N-methylbutylamine	Amine	856		0.29
86	$C_2H_4N_4$	Dicyandiamide	Nitrogen compounds	866		0.29
87	$C_{15}H_{24}$	Germacrene D	Terpenoids	1481	woody, spice	0.28
88	C_9H_{12}	1-Ethyl-3-methylbenzene	Aromatics	957		0.28
89	$C_9H_{18}O$	1-Nonanal	Aldehyde	1105	aldehyde, citrus, orange peel	0.27
90	$C_{15}H_{24}$	Bicyclosesquiphellandrene	Terpenoids	1489		0.27
91	$C_{15}H_{24}$	Beta-guaiene	Terpenoids	1490	sweet, woody, dry, guaiacwood, spicy, powdery	0.27
92	$C_{10}H_{18}O_3$	2-Methylbutanoic anhydride	Others	1190		0.26
93	$C_8H_{12}O$	3,5-Octadien-2-one	Ketone	1073	fruity, green, grassy	0.26
94	$C_8H_{12}O$	3,5-Octadien-2-one	Ketone	1091	fruity, fatty,	0.26

					mushroom	
95	$C_8H_8O_3$	4-Hydroxyphenylacetic acid	Acid	1551		0.24
96	$C_6H_8O_4$	2-Furancarboxylic acid, tetrahydro-3-methyl-5-oxo-	Heterocyclic compound	1304		0.23
97	$C_{10}H_8$	0phthalene	Aromatics	1190	pungent, dry, tarry	0.23
98	$C_{10}H_{18}O_2$	1-Octen-1-ol, acetate	Alcohol	1191		0.22
99	C_7H_6O	BenzAldehyde	Aldehyde	962	sweet, bitter, almond, cherry	0.21
100	C_9H_8S	3-Methylbenzothiophene	Heterocyclic compound	1315		0.21
101	$C_9H_{16}O$	(*Z*)-6-Nonenal	Aldehyde	1104	green, cucumber, melon, cantaloupe, honeydew, waxy, vegetable, orris, violet, leafy	0.21
102	$C_{13}H_{20}O$	4-(4-Methyl-3-pentenyl)cyclohex-3-ene-1-carbaldehyde	Aldehyde	1540	citrus, grassy, aldehydic, floral, marine	0.21
103	$C_{14}H_{30}$	3,5-Dimethyldodecane	Hydrocarbons	1285		0.2
104	$C_5H_{10}O_2$	2-Methylbutanoic acid	Acid	863	pungent, acid, roquefort, cheese	0.2
105	$C_{13}H_{28}$	Tridecane	Hydrocarbons	1300	alkane	0.2
106	$C_8H_{14}O_2$	5-Hydroxyoctanoic acid lactone	Heterocyclic compound	1288	coconut	0.2
107	$C_{10}H_{16}O_2$	3-Methyl-3-(4-methylpent-3-enyl)oxirane-2-carbaldehyde	Aldehyde	1234		0.19
108	$C_4H_3NO_2$	1*H*-Pyrrole-2,5-dione	Heterocyclic	987		0.19

			compound			
109	$C_8H_{16}O$	3.5-Dimethylcyclohexanol	Alcohol	1030		0.19
110	$C_6H_{10}O_2$	5-Methyl-delta-valerolactone	Heterocyclic compound	1095	creamy, fruity, coconut	0.19
111	$C_9H_{16}O$	Trans-4-nonenal	Aldehyde	1105	fruity	0.18
112	$C_7H_{14}O_2$	Hexanoic acid, methyl ester	Ester	925	ethereal, fruity, pineapple, apricot, strawberry, tropical, banana, bacon	0.17
113	$C_{12}H_{25}Br$	2-Bromo dodecane	Haloge0ted hydrocarbons	1505		0.17
114	$C_{10}H_{22}$	Decane	Hydrocarbons	1000	alkane	0.16
115	C_6H_9NOS	5-Thiazoleethanol, 4-methyl-	Heterocyclic compound	1278	fatty, cooked, beefy, juice	0.16
116	$C_{10}H_{16}O$	Hotrienol	Alcohol	1106	sweet, tropical, ocimene, fennel, ginger, myrcene	0.16
117	$C_{12}H_{23}N$	Dodecanenitrile	Nitrogen compounds	1490	citrus, orange, peel, metallic, spicy	0.16
118	$C_{10}H_{16}O$	3,7-Dimethylocta-1,5,7-trien-3-ol	Alcohol	1107	mouldy	0.16
119	$C_8H_{13}NO_2$	Heliotridine	Heterocyclic compound	1495		0.16
120	$C_{16}H_{34}$	Hexadecane	Hydrocarbons	1600	alkane	0.16
121	$C_{17}H_{36}$	Heptadecane	Hydrocarbons	1700	alkane	0.16
122	$C_9H_{20}O$	1-Nonanol	Alcohol	1171	fresh, clean, fatty, floral, rose,	0.15

123	$C_9H_{16}O$	2,6,6-Trimethylcyclohexanone	Ketone	1036	orange, dusty, wet, oily pungent , thujone, labdanum, honey, cistus	0.15
124	$C_{10}H_{20}O_2$	*n*-Amyl isovalerate	Ester	1110	apple, fresh fruit	0.15
125	$C_{10}H_{18}$	2,6-Dimethyl-trans-2,6-octadiene	Hydrocarbons	978		0.15
126	$C_5H_{10}N_2$	5,5-Dimethyl-4,5-dihydro-1*H*-pyrazole	Heterocyclic compound	983		0.15
127	$C_{10}H_{16}O_2$	Lilac aldehyde *D*	Aldehyde	1169	sweet, flowery	0.15
128	$C_{10}H_{16}O_2$	Lilac aldehyde *C*	Aldehyde	1167	sweet, flowery	0.15
129	$C_9H_{16}O_2$	2,2,5-Trimethylhexane-3,4-dione	Ketone	1039		0.14
130	$C_{10}H_{20}O_2$	Butanoic acid, 3-methyl-, 2-methylbutyl ester	Ester	1107	herbal, fruity, earthy, cheese, apple, green	0.14
131	$C_{10}H_{18}O$	2-Isopropyl-5-methylhex-2-enal	Aldehyde	1106	herbal, lavender , woody, green, blueberry, tomato	0.14
132	$C_{10}H_{20}O$	4-Methyl-5-nonanone	Ketone	1116		0.14
133	$C_{10}H_{20}O_2$	3-Methylheptyl acetate	Ester	1118		0.14
134	$C_8H_8O_2$	Benzeneacetic acid	Acid	1262	sweet, honey, floral, honeysuckle, sour, waxy, civet	0.13

135	$C_{13}H_{26}O$	Tridecane	Aldehyde	1513	fresh, clean, aldehydic, soapy, citrus, petal, waxy, grapefruit, peel	0.13
136	$C_8H_{16}O$	(*E*)-2-Octen-1-ol	Alcohol	1068	green, citrus, vegetable, fatty	0.13
137	$C_9H_{16}O_2$	Cyclohexanecarboxylic acid, ethyl ester	Ester	1136	fruity, cheese, winey	0.13
138	$C_{10}H_{20}O$	1-Methyl-4-propan-2-ylcyclohexan-1-ol	Terpenoids	1138		0.13
139	$C_9H_{10}O_3$	Apocynin	Ketone	1493	faint, sweet, vanillin	0.13
140	$C_{14}H_{20}O_2$	Butanoic acid, 1,1-dimethyl-2-phenylethyl ester	Ester	1493	floral, green, herbal, fruity, plum, prune	0.12
141	$C_6H_{10}O$	Cyclohexanone	Ketone	894	minty, acetone	0.12
142	$C_3H_{10}N_2$	1,3-PropanediAmine	Amine	805		0.12
143	$C_{12}H_{20}O_2$	Fenchyl acetate	Ester	1224	fresh, sweet, pine, fir, herbal, citrus	0.12
144	$C_7H_{12}O_2$	2-Propenoic acid, butyl ester	Ester	896		0.12
145	$C_7H_9NO_2$	3-Ethyl-4-methylpyrrole-2,5-dione	Heterocyclic compound	1239		0.12
146	$C_9H_{18}O$	2-Nonen-1-ol	Alcohol	1105	sweet, fatty, melon, cucumber, vegetable	0.12

147	$C_{16}H_{34}$	5,8-Diethyldodecane	Hydrocarbons	1572		0.12
148	$C_{10}H_{18}O$	2,6,6-Trimethylbicyclo[3.1.1]heptan-3-ol	Terpenoids	1179		0.12
149	$C_{11}H_{22}O_2$	Butanoic acid, 3-methyl-, hexyl ester	Ester	1244	sweet, green fruit, apple, unripe apple skin, strawberry	0.11
150	$C_{10}H_{12}O_2$	Acetic acid, 2-phenylethyl ester	Ester	1260	floral, rose, sweet, honey, fruity, tropical	0.11
151	$C_{10}H_{18}O$	1,7,7-Trimethylbicyclo[2.2.1]heptan-2-ol	Terpenoids	1138		0.11
152	$C_7H_{15}NO_2$	2-Propanol, 1-(dimethylamino)-, acetate (ester)	Ester	869		0.11
153	$C_6H_{10}N_2$	2-Propyl-1*H*-imidazole	Heterocyclic compound	1095		0.11
154	$C_9H_{10}O_2$	Paroxypropione	Ketone	1534	phenol	0.11
155	$C_{10}H_{18}O$	(*E*)-2,6-Dimethylocta-5,7-dien-2-ol	Alcohol	1169		0.11
156	$C_9H_{10}O_3$	3,4-Dimethoxybenzaldehyde	Aldehyde	1489	sweet, woody, vanilla	0.1
157	$C_{15}H_{24}$	Ylangene	Terpenoids	1372		0.1
158	$C_9H_{14}O$	2-Amylfuran	Heterocyclic compound	993	fruity, green, earthy, beany, vegetable, metallic	0.1
159	C_5H_7ClO	Cyclobutanecarboxylic acid chloride	Haloge0ted hydrocarbons	853		0.1
160	$C_{11}H_{13}NO_3$	*N*-Benzyloxy-2-carbomethoxyaziridine	Heterocyclic compound	1550		0.1
161	$C_{10}H_{18}O$	1-Decen-3-one	Ketone	1141		0.1
162	$C_{10}H_{16}O$	Isopinocarveol	Terpenoids	1178	woody, warm, balsamic	0.1
163	$C_{13}H_{22}O_2$	3-Cyclohexene-1-methanol, .alpha.,.alpha.,4-trimethyl-, propanoate	Terpenoids	1432	herbal, green,	0.1

					old wood, citrus, geranium, tropical	
164	$C_9H_{16}O$	3-Nonen-5-one	Ketone	1052		0.1
165	$C_{15}H_{24}$	Isoledene	Terpenoids	1375	Odor	0.1
		Total				79.83

(RI: Retention index of compounds on non-polar chromatographic column; Odor: describes the substance's aroma; I: shows the relative abundance of detected VOCs.)
